# Investigation of Long-Term Performance and Deicing Longevity Prediction of Self-Ice-Melting Asphalt Pavement

**DOI:** 10.3390/ma15176026

**Published:** 2022-09-01

**Authors:** Haihu Zhang, Runhua Guo

**Affiliations:** 1 College of Civil Engineering and Architecture, Xinjiang University, Urumqi 830047, China; 2 Department of Civil Engineering, Tsinghua University, Beijing 100084, China

**Keywords:** self-ice-melting asphalt pavement, long-term performance, cycle treatments, salt precipitation, predictive model

## Abstract

Based on laboratory tests, the objective of this study is to assess long-term road performance and to predict deicing longevity of self-ice-melting asphalt pavements containing salt-storage materials. Dry–wet cycles and freeze–thaw cycles were used to treat the specimens at different durations. The long-term road performance of self-ice-melting asphalt mixtures was evaluated by freeze–thaw splitting tests, high-temperature rutting tests, and low-temperature beam bending tests. In addition, the influences of coefficients of void ratio, temperature, vehicle load, crack, and Mafilon (MFL) content on salt precipitation were quantified by conductivity tests, and single consumption of snow and ice melt was quantified by total dissolved solids (TDS) tests. The results show that the long-term water stability, long-term high-temperature stability, and long-term low-temperature crack resistance of self-ice-melting asphalt pavements tended to decrease as the number of dry–wet cycles and freeze–thaw cycles increased. Freeze–thaw cycles exerted deeper influences on the deterioration of road performance than dry–wet cycles, especially on water stability. With increased void ratio and temperature, salt precipitation was accelerated by 1.1 times and 1.5~1.8 times, respectively. Under vehicle loads and cracks, salt precipitation was accelerated by 1.5 times and 1.65 times, respectively. With decreased MFL content, salt precipitation slowed down by 0.54 times. Finally, based on the proportion of each factor relative to the whole life cycle of the pavement, a dicing longevity prediction model was established considering the above factors.

## 1. Introduction

At present, deicing technologies include conductive concrete [1,2,3], circulating heating systems [4,5,6], electric cable heating [7], solar energy [8], geothermal systems [9,10], induction heating, microwave heating [11], etc. Compared with the above deicing technologies, self-ice-melting asphalt pavements with salt-storage additives have the advantages of relatively low cost and easy construction. Therefore, self-ice-melting asphalt pavements containing salt-storage materials are of considerable interest to researchers. The focus of the present research is performance evaluation of self-ice-melting asphalt pavements and how to improve their deicing performance and predict the deicing validity.

It has been proven that the addition of salt-storage additives reduces pavement performance, particularly in terms of water stability [12,13,14,15]. Furthermore, the long-term high-temperature performance, low-temperature performance, and water stability of self-ice-melting asphalt pavements deteriorate with the continuous precipitation of salt during soaking treatment [12,16,17]. Fortunately, the negative impact of salt-storage additives on the pavement performance of asphalt mixtures can be mitigated by adding 0.3% polyester fiber to mixtures [14,16]. The deicing effect of self-ice-melting asphalt pavements depends mainly on the salt precipitation rate, which is mostly influenced by the amount of salt-storage additives [18]. However, increasing the amount of salt-storage additives leads to a decrease in road performance, and salt-storage additives such as Mafilon (MFL) are generally controlled at 4% to 6% of the mass of asphalt mixtures. Therefore, to improve the deicing effect of self-ice-melting asphalt pavements, researchers have attempted to co-integrate self-stressing elastic materials with salt-storage additives into asphalt mixtures either by (a) using high-elasticity modifiers to modify the asphalt and mixing in MFL [19,20] or by (b) using the deformation capacity of rubber particles (RP) to mix MFL and RP jointly into asphalt mixtures [17,21].

For assessment of the deicing longevity of self-ice-melting asphalt pavements, the relationship between salt precipitation and time is obtained mainly by conductivity tests [22], and a mathematical regression model was developed, given the other influencing factors. Zhou et al. [16] established a simple mathematical model for estimating the effective deicing period using three main factors: the total mass of soluble salt, the rate of salt precipitation, and the average number of local annual precipitation days. Zheng et al. [23] developed an evaluation model by introducing an area conversion coefficient, temperature acceleration coefficient, comprehensive influence coefficient, and climate influence coefficient into the regression model of salt precipitation variation with time. Wu et al. [18] set up a predictive model for salt precipitation with the dosage of anti-icing filler, void ratio, rainfall intensity, temperature, and time as independent variables with the help of 1stopt regression analysis software. In addition, Zhang et al. [24] presented a systematic simulation framework for predicting the anti-icing longevity of a thin overlay of asphalt pavement with salt-storage additive. The transport of water and chloride ions in the overlay was simulated by COMSOL finite-element-based software under varying precipitation, temperature, thermal cracking, and fatigue cracking conditions over time.

In summary, the road performance of self-ice-melting asphalt pavements has been investigated, methods to improve deicing effectiveness have been explored and regression models to predict deicing longevity have been proposed. However, the long-term road performance of self-ice-melting asphalt pavements has been less well studied, with existing studies based on all-day immersion [16]. However, self-ice-melting asphalt pavements are in a constant state of alternating between wet and dry and freeze and thaw when in actual application. Freeze–thaw cycles are considered one of the main causes of premature asphalt pavement damage in cold regions [25]. In addition, existing studies have not quantified the accelerating effects of void fraction, temperature, and weather on salt precipitation, although they have taken into account these effects. Furthermore, the mitigating effect of decreasing salt content on salt precipitation and single consumption of snow and ice melt has not been considered. Hence, in the present study, we investigated the long-term road performance of self-ice-melting asphalt after treatment with varying numbers of dry–wet cycles and freeze–thaw cycles. Accordingly, we used conductivity tests and total dissolved solids (TDS) tests to quantify the influence coefficients of each factor and single consumption of snow and ice melt. Finally, a mathematical regression model was established. The results of the present study are intended to provide a reference for the study of long-term road performance and prediction of deicing longevity of self-ice-melting asphalt pavements.

## 2. Materials and Methods

### 2.1. Materials

#### 2.1.1. Asphalt and Aggregate

The basic properties of the SBS-modified asphalt used in this study are shown in Table 1. The aggregates used were all limestones. The technical indicators for aggregates meet the requirements of the Chinese specifications. In addition, in order to prepare asphalt mixtures with an elastic deicing function, some rubber particles (RP) were added to mixtures. The technical indicators of RP are listed in Table 2. The mineral powder used was also limestone powder, which has a density of approximately 2.656 g/cm^3^.

#### 2.1.2. Salt-Storage Additive

**Figure 1:**
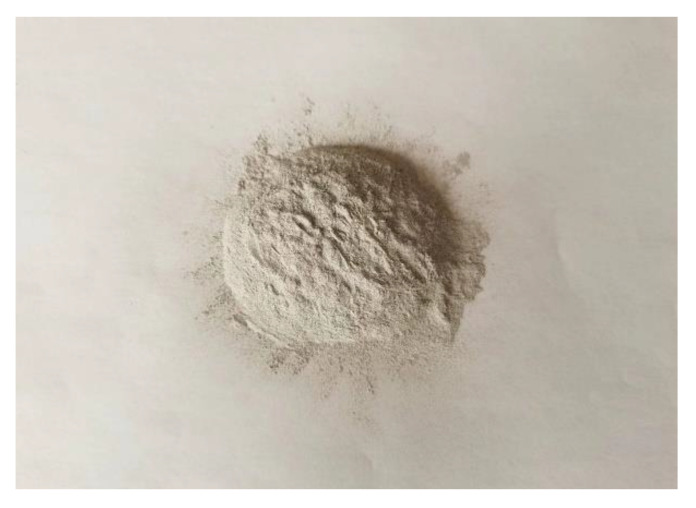
MFL powder.

The salt storage snow melt used in the present study, MFL, was produced by Beijing MFL Frozen Ice Science and Technology Co., Ltd. (Beijing, China), comprising porous volcanic rock and cut into powder, as shown in Figure 1. Its freezing point is −10 °C. The technical specifications of MFL are tabulated in Table 3.

#### 2.1.3. Asphalt Mixture

**Figure 2:**
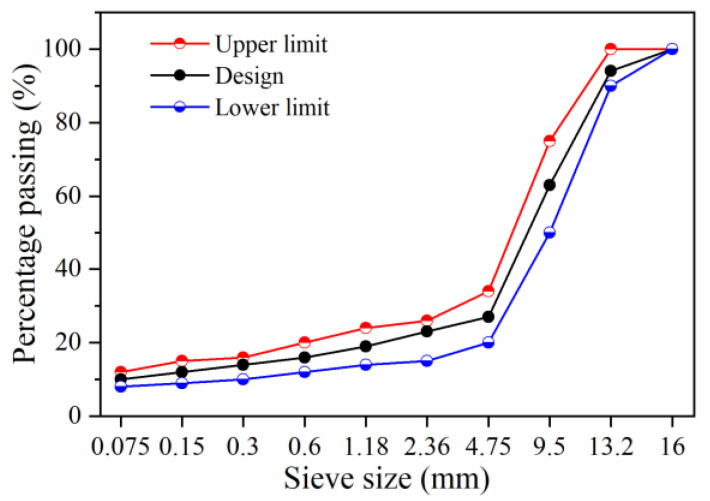
SMA-13 design gradation.

In this research, the self-ice-melting asphalt mixture contained MFL and RP. MFL was added to asphalt mixtures by replacing the mineral powder with equal volume, with a mass fraction of 6%. RP was added by substituting fine aggregates of 1.18~2.36 mm and 2.36~4.75 mm with equal volume, with a mass ratio of RP of 1.18~2.36 mm and 2.36~4.75 mm of 3:1. When the amount of RP is 3% of the total mass of aggregate, asphalt mixtures can meet the requirements of road performance with a satisfactory elastic deicing effect [27]. Therefore, the dosage of RP was set at 3%. Calcium hydroxide (Ca(OH)_2_) interacts with the carboxylic acid in asphalt, which can improve the water stability of the mixture [27,28]. Thus, Ca(OH)_2_ produced by Tianjin Bodi Chemical Co., Ltd. (Tianjin, China) was added to mixtures at a concentration of 1.5% of the total mass of aggregates. Because SMA-13 mixtures exhibit excellent resistance to salt corrosion [29], SMA-13 grading was used in the present study, as shown in Figure 2. The optimum asphalt content was determined to be 6.1% according to the Marshall test. An LDHB-20 vertical fully automatic mixer was used to prepare asphalt mixtures with a heating temperature of 180 °C. The aggregate and RP were stirred for 35 s; then, the asphalt was poured in and stirred for 90 s; finally, the mineral powder, MFL, and Ca(OH)_2_ were added and stirred for 90 s.

### 2.2. Methods

#### 2.2.1. Long-Term Pavement Performance Test

Each asphalt mixture specimen was subjected to dry–wet cycles and freeze–thaw cycles of 3, 7, and 14 times, respectively. Here, one dry–wet cycle was defined as follows. The specimens were immersed in water at 20 °C for 12 h and placed in the laboratory for 12 h. One freeze–thaw cycle was defined as follows. The specimens were frozen at −18 °C for 16 h and immersed in water at 20 °C for 8 h. Subsequently, a freeze–thaw splitting test, rutting test (60 °C), and trabecular bending test (−10 °C) were carried out on asphalt mixtures according to methods T 0729, T 0719 and T 0715 in of the Chinese test specification (JTG E20-2011) [26]. The freeze–thaw splitting test strength ratio (*TSR*), dynamic stability (*DS*), and maximum flexural tensile strain (*ε_B_*) were used as indicators to evaluate the water stability, high-temperature stability, and low-temperature crack resistance of asphalt mixtures, respectively.

#### 2.2.2. Salt Precipitation Test

**Figure 3:**
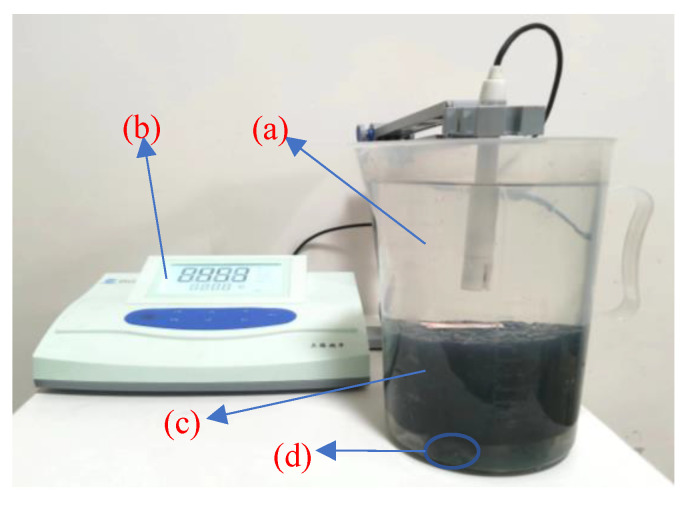
Diagram of salt precipitation test by full dissolution method: (**a**) 1500 mL drinking water; (**b**) DDS-11A conductivity meter; (**c**) Marshall specimen; (**d**) three stones.

The main methods used to determine salt precipitation are the full dissolution method and the single-surface dissolution method. However, when the single-surface dissolution method is used, the salt precipitation rate is slow, resulting in inaccurate measurement results [23]. Therefore, we applied the full dissolution method, as shown in Figure 3. The specimens were kept at a temperature of 20 °C in a constant temperature curing cabinet during the test as the basis for subsequent tests on salt precipitation in specimens with varying void ratios, temperatures, and MFL content, and in the presence or absence of vehicle load and cracking. Specific test descriptions are presented below.

(1) Salt precipitation at varying void ratios

In our study of salt precipitation at varying void ratios, the number of double-sided compactions of the Marshall specimens was 50 and 75 respectively. The average void ratios were measured as 5.2% and 3.8%, respectively. The conductivity of the solution was measured for 168 h. The test interval was 1 h for the first 12 h and then every 12 h.

(2) Salt precipitation at varying temperatures

Salt precipitation losses are most severe during summer rainfall [12]. Therefore, we focused on the effect of increasing temperature on salt precipitation. Marshall specimens were tested for salt precipitation at 20 °C, 30 °C, and 40 °C. A low-temperature constant temperature tank was used to heat the specimens in a water bath to maintain the test temperature. The conductivity of the solution was measured for 12 h, and the test interval was 1 h.

(3) Salt precipitation under vehicle load

**Figure 4:**
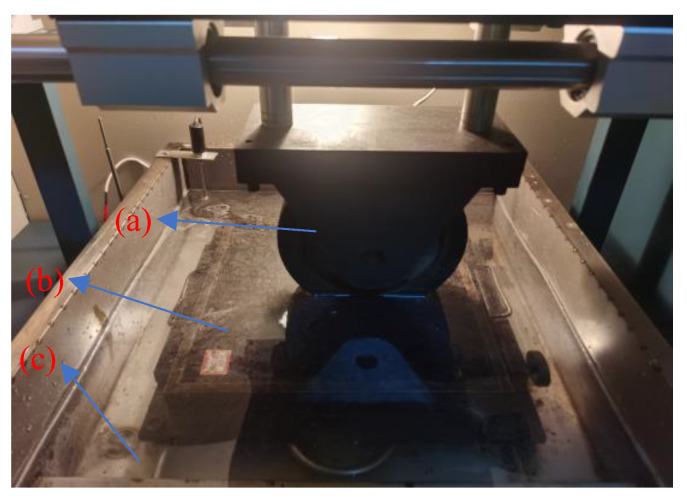
Diagram of salt precipitation test under vehicle load: (**a**) vehicle load; (**b**) rutting plate specimen; (**c**) 20 L drinking water.

Figure 4 depicts the salt precipitation test under vehicle load with a traffic load applied using an LDCZ-5 automatic rutting tester. In the first 6 h, the conductivity of the solution was tested without vehicle load. In the last 6 h, the rut tester started to test the conductivity of the solution under a vehicle load. The test interval was 1 h.

(4) Salt precipitation under cracking

**Figure 5:**
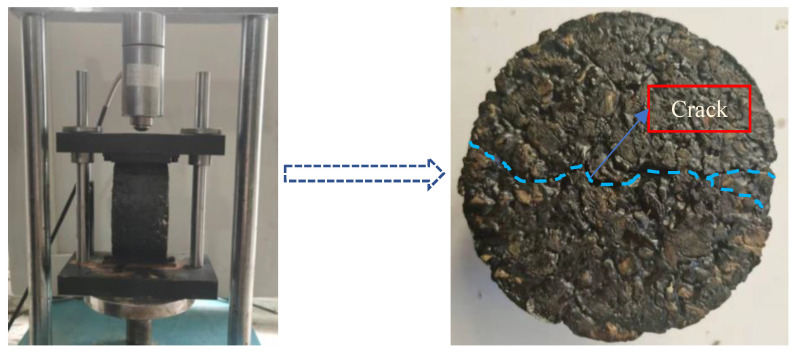
Marshall specimen with crack.

As shown in Figure 5, the Marshall specimen was cracked by applying a load to it using an asphalt mixture stability tester. The test operation was the same as that shown in Figure 3, and the test time was the same as that for salt precipitation at varying void ratios.

(5) Salt precipitation with varying MFL contents

As the MFL content of a self-melting snow pavement continues to decrease during actual service, we tested salt precipitation when the MFL content was reduced by 50%. This means that the MFL content of the Marshall specimen was 3%. The test operation was the same as that shown in Figure 3. The conductivity of the solution was measured for 720 h. The test interval was 1 h for the first 12 h and then every 12 h.

#### 2.2.3. Single-Consumption of Snow and Ice Melt Test

**Figure 6:**
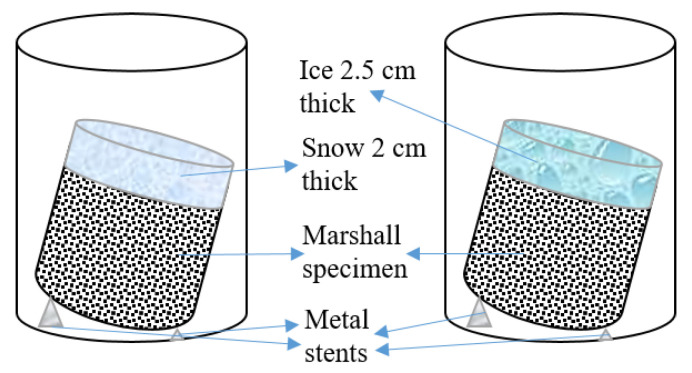
Diagram of single consumption of snow and ice melt test.

The test setup shown in Figure 6 was placed into a low-temperature test chamber and maintained at a temperature of −1 °C. The solution was collected after all the snow and ice had melted and tested for volume and TDS using a measuring cylinder and DDS-11A conductivity meter, respectively. The high salt content of the ice melt solution caused the TDS to exceed the range of the DDS-11A conductivity meter. Therefore, to measure the TDS of the melted ice solution, 10 mL of the sample solution was diluted with 30 mL of pure water.

**Figure 7:**
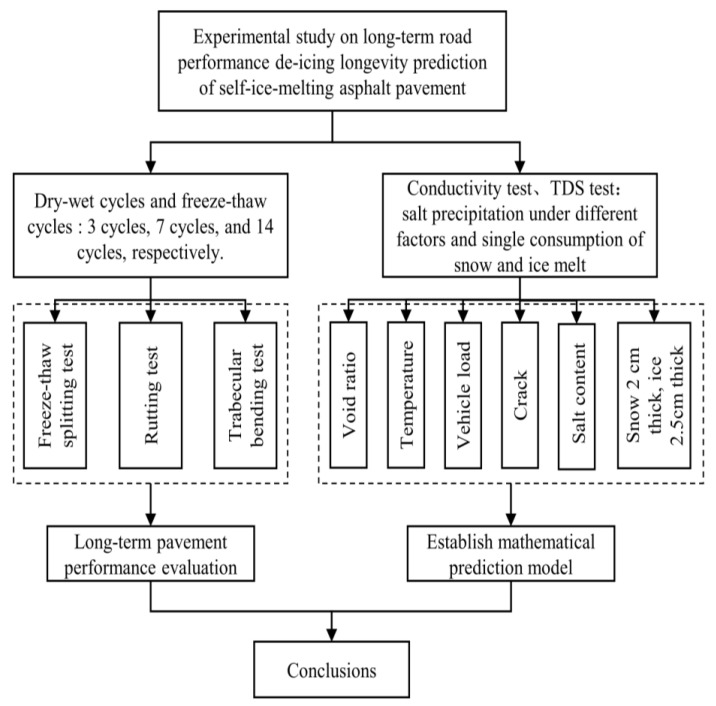
Schematic diagram of research route.

The research route employed in the present study is shown in Figure 7.

#### 2.2.4. Data Processing Methods

**Figure 8:**
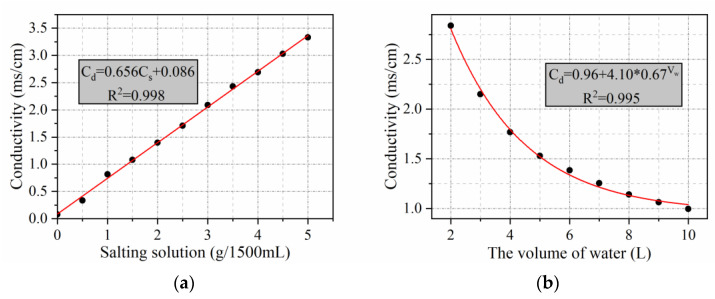
The conductivity of the solution as a function of salt content and water volume: (**a**) relationship between solution conductivity and salt content; (**b**) relationship between solution conductivity and water volume.

**Figure 9:**
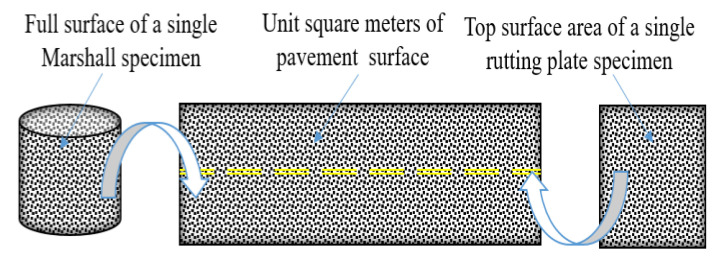
Area conversion schematic diagram.

To visualize the amount of salt precipitation, the conductivity data measured by the Marshall and rutting plate specimens were converted into the mass of salt precipitation per unit square meter of pavement. As indicated in Figure 8, the conductivity of the solution is linearly related to the MFL content of the solution when the amount of water is certain; when the MFL content of the solution is certain, the conductivity of the solution is represented by a curved function of the amount of water added. Based on the above functional relationship, the conductivity can be converted into the mass of salt precipitated in 1500 mL of solution. As shown in Figure 9, the salt precipitation per unit square meter of pavement can be converted using Equation (1).

(1) $$\begin{array}{l} S_{1}=(2\pi r^{2}+2\pi rh)\times 10^{-6}\\ S_{2}=b\times l\times 10^{-6} \end{array}$$

where *S*_1_ is the full surface area of the Marshall specimen, (m^2^); *r* is the radius of the Marshall specimen, which is 101.6/2 mm; *h* is the height of the Marshall specimen, which is 63.5 ± 1.3 mm; *S*_2_ is the top surface area of the rutting plate specimen, (m^2^); *b* is the length of the rutting plate specimen, which is 300 mm; and *l* is the width of the rutting plate specimen, which is 300 mm.

## 3. Results and Discussion

### 3.1. Evaluation of Long-Term Pavement Performance

**Figure 10:**
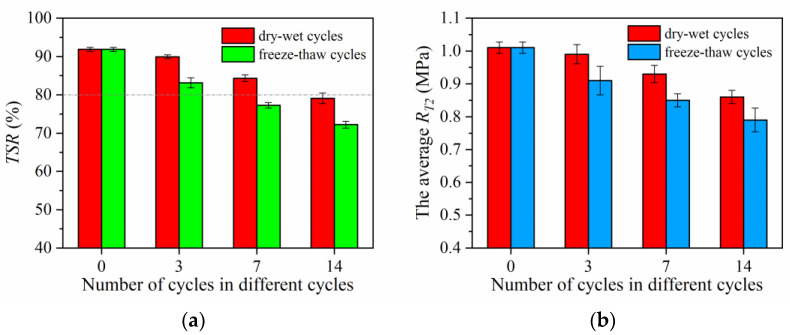

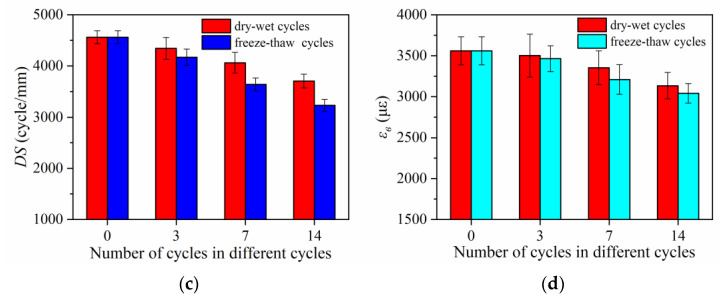
Long-term pavement performance test results under varying numbers of cycles: (**a**) freeze–thaw splitting tensile strength ratio; (**b**) average splitting tensile strength; (**c**) dynamic stability; (**d**) maximum flexural tensile strain.

Figure 10 shows the long-term pavement performance of self-ice-melting asphalt mixture after dry–wet cycles and freeze–thaw cycles. As shown in Figure 10, the *TSR*, $\bar{R}$*_T_*_2_, *DS*, and *ε_B_* of asphalt mixtures decrease as the number of treatments increases in both cycles. To quantify this downward trend, Table 4 shows the specific decrease in performance with the number of cycles in both dry–wet and freeze–thaw cycles. Figure 10 and Table 4 show that the *TSR*, $\bar{R}$*_T_*_2_, and *DS* decreased more than *ε_B_* as the number of cycles increased. Furthermore, each property deteriorates more with an increased number of freeze–thaw cycles than with an increased number of dry–wet cycles. *TSR* does not meet the specification of *TSR* ≥ 80% [30] when the number of dry–wet cycles reaches 14 or when the number of freeze–thaw cycles reaches 7.

In summary, the water stability, high-temperature performance, and low-temperature performance of self-ice-melting pavement show varying degrees of downward trend with the extension of the number of dry–wet cycles and freeze–thaw cycles, with tendency for the performance indicators to decrease more significantly during freeze–thaw cycles than dry–wet cycles. From the perspective of asphalt materials, this can be explained as follows: When asphalt comes into contact with ice at low temperatures, the difference in concentration of surface and internal chemicals promotes the entry of water into the asphalt voids, resulting in a change in the composition ratio of the asphalt material [31,32]. Sulfur oxides in asphalt also increase at low temperatures [33], making it brittle and hard. In addition, ice conversion vapor promotes the oxidation reaction of asphalt in the thawing phase, which oxidizes the active hydrocarbons in asphalt to carboxylic acids [33]. This reaction can enhance the hydrophilic nature of the asphalt. Furthermore, as the number of freeze–thaw cycles increases, SBS degrades and loses its cross–linked network [34,35].

**Figure 11:**
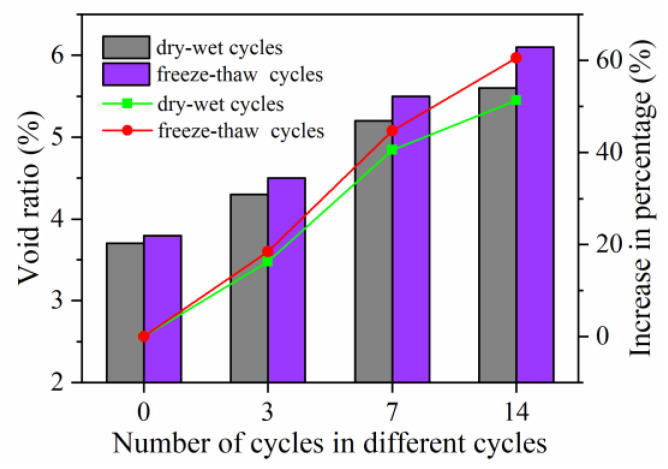
Variation in void ratio with the number of cycles under varying numbers of cycles.

Freeze–thaw cycles cause damage to the internal structure of asphalt mixtures [36,37]. This is reflected in a significant increase in the size and number of internal pores [38], which increases the path of movement of moisture within asphalt mixtures [39], making it easier to enter the asphalt and the asphalt–aggregate interface. Furthermore, freeze–thaw cycles increase the vertical flow penetration of asphalt mixtures and exacerbate the seepage anisotropy in asphalt mixtures [40]. Water flows more easily through asphalt mixtures and the scouring effect of water on the internal structure of the asphalt mix is more pronounced [41]. As shown in Figure 11, the void ratio increases as the number of cycles increases, and with freeze–thaw cycles further increasing the void ratio. This may also be the reason why the road performance of asphalt mixtures is reduced considerably more under freeze–thaw cycles than under dry–wet cycles. Moreover, micro–cracks appear inside asphalt mixtures under the action of freezing and swelling forces, which further extend and expand under the action of repeated freezing and swelling forces, resulting in a gradual accumulation of freeze–thaw damage in asphalt mixtures [42]. In addition, under the effect of frost heave, the binder force and the internal wear resistance of asphalt mixtures are reduced, resulting in a decrease in mechanical properties and a reduction in the stiffness and strength of the mixture [43].

Although the high- and low- temperature performance of asphalt mixtures degrades with an increase in the number of both cycles, it still meets the functional requirements of pavement. In addition, asphalt mixtures age to a certain extent during actual road service, which improves the high-temperature stability of the pavement [16]. Therefore, there is no need to excessively considering the effects of dry–wet cycles and freeze–thaw cycles on the high- and low- temperature performance of self-ice-melting pavements. However, a method is required to enhance the water stability, and the *TSR* value of 14 dry–wet cycles or 7 freeze–thaw cycles should be highlighted as an evaluation indicator. We suggest the use of highly–elastic modified asphalt or high-viscosity modified asphalt. In particular, highly–elastic modified asphalt not only benefits the performance of pavements but also improves their elastic deicing performance. However, highly–elastic modified asphalt is not effective in improving water stability; therefore, Ca(OH)_2_, anti-peeling agents, etc., can be added to mixtures to improve water stability.

### 3.2. Establishment of a Long-Term Predictive Model

#### 3.2.1. Basic Assumptions

To design a simple and effective model that eliminates the effects of certain uncertainties, the following assumptions need to be made before establishing the model:

(1) The salt dissolved from the Marshall piece is equal to the salt dissolved from the unit area of the pavement without other losses [13];

(2) The precipitated salts are completely dissolved in water solutions as solvents and dissolved into water as ice melts and snow melts in winter;

(3) The melting of snow and ice is entirely the result of the action of precipitated salts, irrespective of the contribution of other energies (e.g., geothermal heat, wheel friction, vehicle exhaust, and rolling).

#### 3.2.2. Original Model

**Figure 12:**
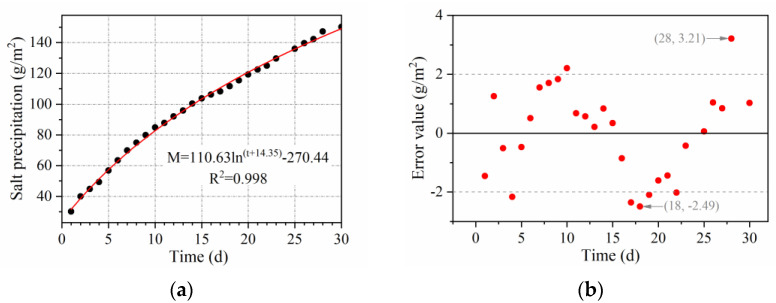
Variation in salt precipitation over time and mathematical fitting error: (**a**) salt precipitation over time; (**b**) error between the predicted value and measured value.

Figure 12a shows the test results of salt precipitation over time under natural immersion. A mathematical fit to the data was found to fit the natural logarithmic function well, as shown in Equation (2).

(2) $$S_{p}=110{.63\ln}^{(t+14.5)}-270.44$$

where *S_p_* is salt precipitation (g/m^2^), and *t* is the soaking time (days).

Figure 12b shows the error distribution between the predicted and measured values. As shown in Figure 12b, the majority of the error values lie at ±2 g/m^2^, with the largest at 3.21 g/m^2^. The mathematical model satisfactorily characterizes the variation in salt precipitation over time.

#### 3.2.3. Void Ratio Influence Coefficient

**Figure 13:**
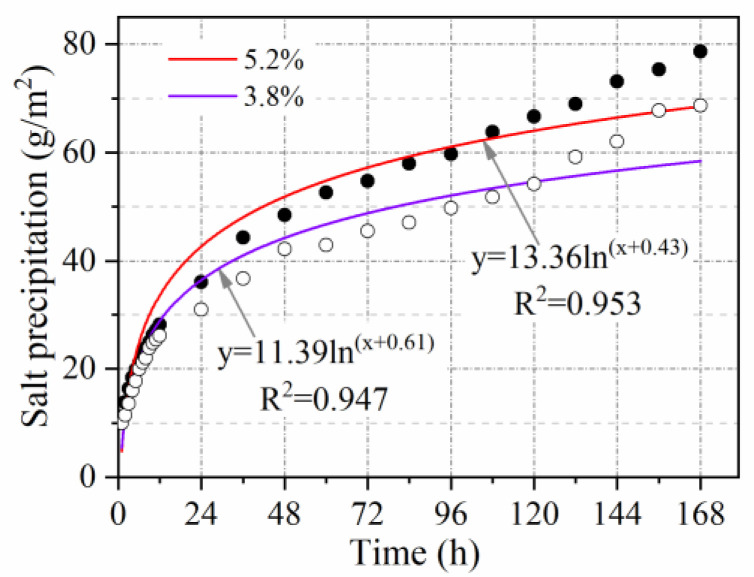
Effect of void ratio on salt precipitation.

The salt precipitation at varying void ratios is plotted in Figure 13. The results indicate that an increase in void ratio leads to increased salt precipitation. Because the increase in voids makes it easier for water to enter the pavement through the voids. As a result, MFL in self-ice-melting asphalt pavement is dissolved and precipitated continuously under the action of diffusion mass transfer. Mathematical fitting shows that salt precipitation is around 1.1 times higher at a void ratio of 5.2% than at a void ratio of 3.8%. Therefore, the void ratio influence coefficient is considered to be 1.1.

#### 3.2.4. Temperature Influence Coefficient

**Figure 14:**
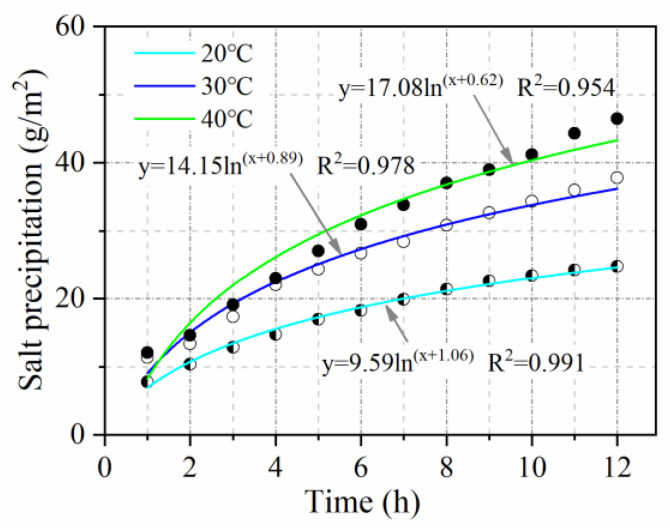
Effect of temperature on salt precipitation.

The pattern of salt precipitation over time when the temperature was 20 °C, 30 °C, and 40 °C is shown in Figure 14. With increased temperature, salt precipitation increases. As the increase in temperature accelerates the movement of molecules. Furthermore, the increase in temperature causes the asphalt mixtures to soften, which reduces the binding of MFL, resulting in faster salt precipitation. Compared with salt precipitation at 20 °C, salt precipitation at 30 °C and salt precipitation at 40 °C are increased by approximately 1.5 and 1.8 times, respectively. Therefore, the average temperature influence coefficient is regarded as 1.65.

#### 3.2.5. Vehicle Load Influence Coefficient

**Figure 15:**
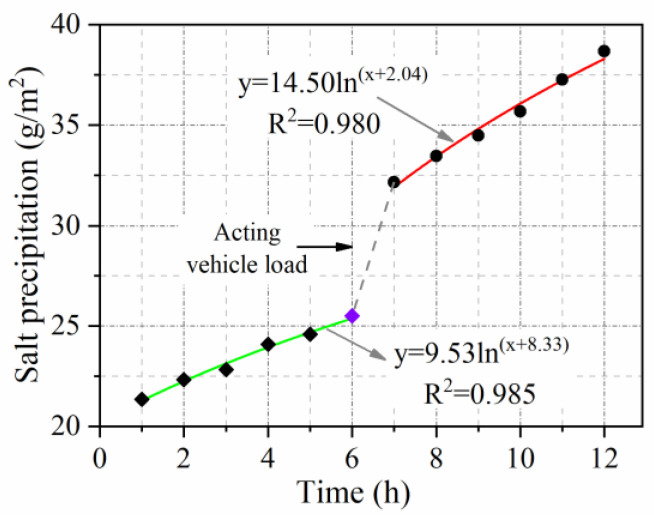
Effect of vehicle load on salt precipitation.

Figure 15 presents the regularity of salt precipitation over time in the presence and absence of vehicle load. As shown in Figure 15, when a vehicle load was applied at the 6th hour after the start of the measurement, there was a significant increase in salt precipitation compared with the non-load period. Due to the pumping action caused by the vehicle load. Salt precipitation with vehicle load is almost 1.5 times that achieved without vehicle load. Hence, the vehicle load influence coefficient can be deemed to be 1.5.

#### 3.2.6. Crack Influence Coefficient

**Figure 16:**
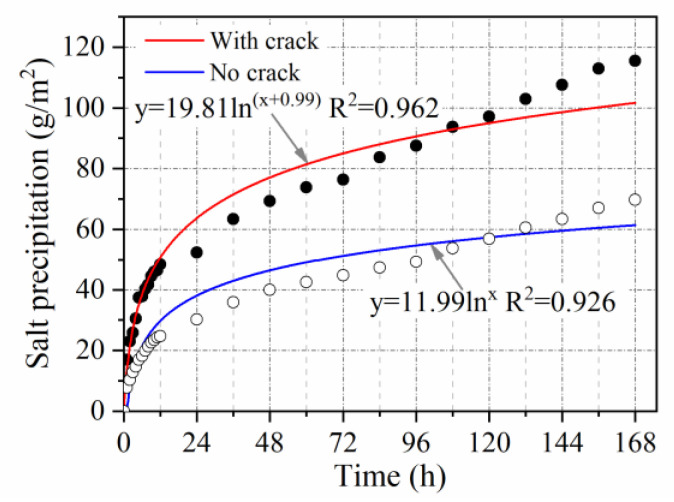
Effect of cracks on salt precipitation.

Figure 16 shows the time dependence of salt precipitation with and without cracks. Salt precipitation increases significantly with the development of cracks because water can more easily enter the interior of pavement through cracks, leading to a transfer of the loss of MFL from capillary action to dissolution. Salt precipitation in pavement with cracks is approximately 1.65 times greater than that in pavement without cracks. As such, the crack influence coefficient can be taken to be 1.65.

#### 3.2.7. Salt Content Influence Coefficient

**Figure 17:**
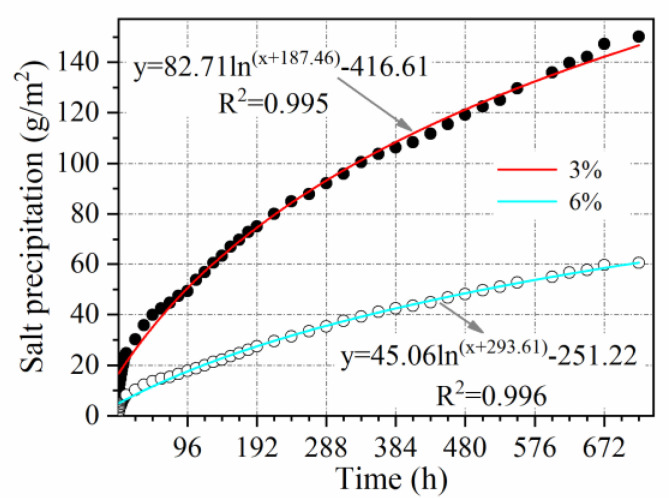
Effect of salt content on salt precipitation.

Figure 17 illustrates the evolution of salt precipitation over time as the salt content is reduced from 6% to 3%. With the use of self-ice-melting pavements, the salt content in the pavement continues to decrease, resulting in a slow–down in the rate of salt precipitation. Due to the decrease in solute concentration gradient, leading to the attenuation of the diffusion mass transfer effect. Salt precipitation at a salt content of 3% is only roughly 0.54 times as much as salt precipitation at a salt content of 6%. Accordingly, the coefficient of influence of salt content can be calculated as 0.54.

#### 3.2.8. Original Model Modification

As demonstrated in Section 3.2.3, Section 3.2.4, Section 3.2.5, Section 3.2.6 and Section 3.2.7, it is evident that void ratio, temperature, vehicle load, and cracks have an accelerating effect on salt precipitation. In contrast, the salt precipitation rate decreases in the later stages due to the decrease in salt content. Therefore, to make the prediction model more realistic, the above influencing parameters need to be inserted to calibrate the model. In the present study, the combination of parameters was based on the proportion of each factor in the life cycle of the pavement. The parameters are introduced below.

(1) According to the preliminary void ratio tests, the void ratio increases as the number of D-WC and F-TC increases. The void ratio increases in the short term when the pavement is actually in use. Therefore, the void ratio acceleration coefficient (*V*) is applied throughout the life cycle of the pavement. Therefore, *V* = 1.1.

(2) Temperature acceleration is mainly considered during summer rainfall and throughout the life cycle of the pavement. Let the average number of days of rainfall in a city throughout the year be *d*_1_, of which the proportion of days of rainfall in summer is *α*. These parameters can be obtained from city meteorological data. The temperature influence coefficient can then be calculated with Equation (3) as:

(3) $$T=\frac{\alpha \cdot d_{1}}{365}\times 1.65+1$$

where *T* is the temperature influence coefficient, *α* is the proportion of summer rainfall days to annual rainfall days, and *d*_1_ is the number of rainfall days in a year (days).

(3) During the actual service of the road, the vehicle load is applied to the whole life cycle of the pavement. Therefore, the vehicle load influence coefficient (*L*) is 1.5.

(4) As cracks develop after a certain number of years of pavement service, they run through the entire life cycle of the pavement. Here, we applied a discount coefficient to represent this case, denoted as *β*. We assumed that cracks are present during 80% of the full life cycle of the pavement. Thus, the crack influence coefficient can be determined by Equation (4).

(4) C=β×1.65

where *C* is the crack influence coefficient, and *β* is the discount coefficient, which is 0.8.

(5) The salt content is in a state of decline during the entire life cycle of the pavement. In this study, the effect of salt content on salt precipitation is expressed in terms of *γ*, which takes values ranging from 0.54 to 1. To simplify the calculation, the median value (0.77) is taken as the calculated value in this paper.

In summary, the original model can be modified by Equation (5).

(5) $$S_{q}=(V\cdot T\cdot L\cdot C\cdot \gamma )\times [110{.63\ln}^{(t+14.5)}-270.44]\;$$

where *V* is the void ratio acceleration coefficient, which is 1.1; *T* is the temperature influence coefficient; *L* is the vehicle load influence coefficient, which is 1.5; *C* is the crack influence coefficient, which is 1.32; and *γ* is the salt content influence coefficient, which is 0.77.

### 3.3. Snow and Ice Melt Consumption

#### 3.3.1. Single Consumption of Snow and Ice Melt

Table 5 shows the results of the salt consumption test for a single snow and ice melt. Accordingly, 22 mg of salt is required to melt 2 cm of snow on 81.032 cm^2^ of pavement, which means that 27 g of salt is required to melt 2 cm of snow per square meter of pavement. It can also be estimated that 10 mL of ice-melting solution contains 38.56 mg of salt, which means that 81.032 cm^2^ of pavement requires 767 mg of salt to melt 2.5 cm of ice. An estimated 95 g of salt is consumed per square meter of road surface to melt 2.5 cm of ice. In addition, some salt may remain on the surface of the specimen during the melting of the snow and ice, resulting in a lower estimated value than the actual value.

#### 3.3.2. Annual Consumption of Snow and Ice Melt

(1) According to the China Meteorological Administration’s (CMA) classification of snowfall levels, light snow is defined as snow on the ground at a depth of less than 3 cm, moderate snow is defined as snow on the ground at a depth of 3 to 5 cm, and heavy snow is defined as snow on the ground at a depth equal to or greater than 5 cm. To simplify the model, in the present study, we assumed a moderate snow standard, which means that the snow depth on the ground is 3 cm. The average number of snow days in a year can be identified based on local meteorological data and denoted as *S*. Therefore, the amount of salt required to melt snow per unit square meter of pavement in a year can be calculated by Equation (6).

(6) Msnow=3S×272=40.5S

where *M_snow_* is the salt consumption for snow melting (g/m^2^), and *S* is the average number of snowfall days a year (days).

(2) The number of times the road freezes each year is denoted by *I*, which can be determined from local meteorological data. According to traffic meteorological data, generally, the thickness of ice on the road surface is 0.5 cm to 1 cm; in the present study, it was assumed to be 0.75 cm. Therefore, the amount of salt required to melt ice per unit square meter of pavement in a year is determined by Equation (7).

(7) Mice=0.75I×952.5=28.5I

where *M_ice_* is the salt consumption for ice melting (g/m^2^), and *I* is the average number of ice formations per year (time).

In summary, when salt consumption of melting ice and snow is considered, the annual salt loss per square meter can be calculated by Equation (8). The ultimate mathematical prediction model is as follows:

(8) $$\begin{array}{ll} M & =S_{q}+M_{snow}+M_{ice}\\ & =(V\cdot T\cdot L\cdot C\cdot \gamma )\times [110{.63\ln}^{(t+14.5)}-270.44]+(40.5S+28.5I) \end{array}$$

where *M* is the annual salt precipitation (g/m^2^).

### 3.4. Application of Prediction Model

Taking Beijing, China, as an example, a check of Beijing meteorological data shows that the average number of days of rainfall in Beijing is 73.9 d throughout the year, of which 70%~76% occur in summer. In this paper, we assume that 75% of rainfall days occur in summer. The average number of snowy days in Beijing is 10.3 d. Winter icing occurs mainly in December and January, 1 and 5.7 times, respectively. The number of icing events in November, February, and March is based on the maximum number of icing in November, which is 1.8 [44]. Therefore, *T* = 1.15, *S* = 10.3 days, and *I* = 8.5 times can be determined. Salt precipitation per unit square meter of pavement for one year can be calculated by Equation (9).

(9) $$\begin{array}{ll} M & =(V\cdot T\cdot L\cdot C\cdot \gamma )\times [110{.63\ln}^{(t+14.5)}-270.44]+(40.5S+28.5I)\\ & =1.1\times 1.15\times 1.5\times 1.32\times 0.77\times [110{.63\ln}^{(365+14.5)}-270.44]\;\\ & +(40.5\times 10.3+28.5\times 8.5)\\ & =1404{.96\;(g/m}^{2}) \end{array}$$

In this study, the asphalt content of self-ice-melting asphalt pavement was 6.1%, with a density of 2.446 g/cm^3^. MFL dosage accounts for 6% of the total mass of aggregate. We assumed a pavement thickness of 5 cm. Therefore, the total MFL mass per unit square meter of self-ice-melting asphalt pavement is calculated to be 6890.38 g. Therefore, the deicing longevity of self-ice-melting asphalt pavement in Beijing is 4.9 years. In addition, the internal stress of RP causes the snow and ice on the pavement to break up and melt under vehicle load [45], which extends the deicing longevity of self-ice-melting asphalt pavement.

## 4. Conclusions

In this paper, the long-term pavement performance of self-ice-melting asphalt pavement was studied according to dry–wet cycles and freeze–thaw cycles. The influence coefficients of void ratio, temperature, vehicle load, crack, and salt content on salt precipitation were quantified by conductivity tests. The single consumption of snow and ice melt was tested by TDS tests. Finally, the deicing longevity of self-ice-melting asphalt pavement was established based on mathematical regression fitting. The main conclusions are as follows:

1. The long-term water stability, long-term high-temperature stability, and long-term low-temperature crack resistance of self-ice-melting asphalt pavements are reduced by 14.19%, 18.79%, and 11.96%, respectively, under dry–wet cycles and by 21.35%, 29.18%, and 14.57%, respectively, under freeze–thaw cycles. With respect to long-term water stability, with 14 dry–wet cycles or 7 freeze-thaw cycles, the *TSR* is less than 80%.
2. The relationship between salt precipitation under natural immersion and time follows a logarithmic function (R^2^ > 0.9). Salt precipitation is accelerated by void ratio, temperature, vehicle load, and cracking, with accelerating factors of *V* = 1.1, *T* = 1.65, *L* = 1.5, and *C* = 1.65, respectively. Salt precipitation slows down with decreasing salt content and is only 0.54 times the original salt precipitation when the salt content is reduced to half.
3. Based on the proportion of each factor in the life cycle of pavement, the influence coefficients of void ratio, temperature, vehicle load, and cracking are incorporated into the mathematical regression model of salt precipitation over time. Furthermore, the prediction model of deicing longevity of self-ice-melting asphalt pavement is established considering the single consumption of snow and ice melt.

Dry–wet cycles and freeze–thaw cycles deteriorate the long-term road performance of self-melting asphalt pavements, which should be taken into account in future research. In addition, the compaction degree and salt content of pavements should be reasonably controlled, and cracks should be repaired in time to extend the deicing longevity of pavement.

## Figures and Tables

**Table 1:** Basic properties of SBS-modified asphalt.

Technical Indicator		Measured Value	Standard Value	Test Method [26]
Penetration at 25 °C (0.1 mm)		55.0	40–60	T 0604
Penetration index (PI)		0.16	≥0	T 0604
Softening point TR&B (°C)		80.6	≥60	T 0606
Ductility 5 °C (cm)		33.0	≥20	T 0605
After TFOT	Quality change (%)	0.59	≤1.0	T 0609
	Penetration ratio 25 (%)	70	≥65	T 0604
	Ductility at 5 °C (cm)	26.4	≥15	T 0605

**Table 2:** Technical indicators of RP.

Technical Indicator	Value	Specification
Apparent relative density (g/cm^3^)	1.05	≤1.25
Water content (%)	0.36	≤0.75
Fiber content (%)	0.34	≤0.75
Slender and flat particle content (%)	6	≤10

**Table 3:** Technical indices of MFL.

Technical Indicator	Value	Specification
Density (g/cm^3^)	2.27	2.25–2.35
Salt precipitation (%)	≤0.4	≤0.4
PH value	8.23	8.0–8.5
Moisture absorption rate	0.5	≤0.7
Main components	NaCl, CaCO_3_, Fe_2_O_3,_ etc.	—

**Table 4:** Decrease rate of each performance index with the number of dry–wet cycles and freeze–thaw cycles.

Performance Indicator	Decline Rate of Each Performance Index with the Number of Cycles (%)					
	3 Cycles		7 Cycles		14 Cycles	
	D-WC	F-TC	D-WC	F-TC	D-WC	F-TC
*TSR*	2.08	9.48	8.18	15.88	14.19	21.35
$\bar{R}$ *_T_*_2_	1.98	9.90	7.92	15.84	14.85	21.78
*DS*	4.78	8.56	10.92	20.19	18.79	29.18
*ε_B_*	1.66	2.67	5.84	9.83	11.96	14.57

Note: “dry–wet cycles” and “freeze–thaw cycles” are abbreviated as “D-WC” and “F-TC”, respectively.

**Table 5:** Single snow- and ice- melt test results.

Type of Test	TDS (mg/L)			Average TDS (mg/L)	Average Volume (mL)
Snow melting	758	684	660	700.67	31.4
Ice melting	968	951	973	964	198.8

## Data Availability

Not applicable.
